# Construction of pH-sensitive Her2-binding IgG1-Fc by directed evolution

**DOI:** 10.1002/biot.201300483

**Published:** 2014-08-02

**Authors:** Michael W Traxlmayr, Elisabeth Lobner, Christoph Hasenhindl, Gerhard Stadlmayr, Chris Oostenbrink, Florian Rüker, Christian Obinger

**Affiliations:** 1 Christian Doppler Laboratory for Antibody Engineering at Department of Chemistry and Department of Biotechnology, BOKU – University of Natural Resources and Life SciencesVienna, Austria; 2 Department of Chemistry, Vienna Institute of BioTechnology, BOKU – University of Natural Resources and Life SciencesVienna, Austria; 3 Department of Biotechnology, Vienna Institute of BioTechnology, BOKU – University of Natural Resources and Life SciencesVienna, Austria; 4 Department of Material Sciences and Process Engineering, Institute of Molecular Modeling and Simulation, BOKU – University of Natural Resources and Life SciencesVienna, Austria

**Keywords:** Antibody engineering, Directed evolution, Fcab, pH-depending binding, Yeast surface display

## Abstract

For most therapeutic proteins, a long serum half-life is desired. Studies have shown that decreased antigen binding at acidic pH can increase serum half-life. In this study, we aimed to investigate whether pH-dependent binding sites can be introduced into antigen binding crystallizable fragments of immunoglobulin G1 (Fcab). The C-terminal structural loops of an Fcab were engineered for reduced binding to the extracellular domain of human epidermal growth factor receptor 2 (Her2-ECD) at pH 6 compared to pH 7.4. A yeast-displayed Fcab-library was alternately selected for binding at pH 7.4 and non-binding at pH 6.0. Selected Fcab variants showed clear pH-dependent binding to soluble Her2-ECD (decrease in affinity at pH 6.0 compared to pH 7.4) when displayed on yeast. Additionally, some solubly expressed variants exhibited pH-dependent interactions with Her2-positive cells whereas their conformational and thermal stability was pH-independent. Interestingly, two of the three Fcabs did not contain a single histidine mutation but all of them contained variations next to histidines that already occurred in loops of the lead Fcab. The study demonstrates that yeast surface display is a valuable tool for directed evolution of pH-dependent binding sites in proteins.

## 1 Introduction

Therapeutic proteins such as antibodies, cytokines, hormones, or growth factors are increasingly important for the treatment of a variety of diseases. For most of these proteins, a long in vivo half-life is desired. However, many proteins are rapidly cleared from the serum, which may have negative impacts on required dosage, administration intervals, as well as on the therapeutic potential of the drug [1].

Several mechanisms have been shown to decrease serum half-life. One of them is receptor-mediated endocytosis that internalizes many receptors at high rates. If a therapeutic protein binds to such a receptor, it will be internalized as well and located in sorting endosomes at a pH of ∼6 [2]. From there, the proteins are either delivered to late endosomes and, finally, to lysosomes for degradation, or to the plasma membrane for recycling. Interestingly, it has been demonstrated that the half-life of antibodies recognizing the IL-6 receptor [3] or proprotein convertase subtilisin kexin type 9 (PCSK9) [4] can be increased by engineering these antibodies for decreased antigen-binding at acidic pH. The authors proposed that reduced affinity at pH 6.0 results in release from the internalized receptor in the endosome and subsequent recycling to the cell surface instead of degradation in the lysosome. This strategy was also successful for extending the half-life of granulocyte colony-stimulating factor [5]. In the case of antibodies, it has also been shown that this recycling process is dependent on the neonatal crystallizable fragment (Fc) receptor (FcRn) [4]. Thus, the antibody seems to dissociate from the receptor in the endosome and binds to FcRn, which delivers it back to the cell surface.

Together, these studies clearly show the beneficial effect of a pH-dependent binding site in antibodies, which bind to receptors with high turnover rates. This underlines the importance of highly efficient and rapid methods for engineering a pH-sensitive binding site in therapeutic proteins. In most of the studies, pH-dependence was achieved by introducing histidines into the binding-site [3–6]. More recently, directed evolution by using yeast display has also been employed for engineering pH-dependent binding sites in the hyperthermophilic protein Sso7d [7] by selecting for binding at neutral pH and reduced binding at acidic pH.

**Figure 1:**
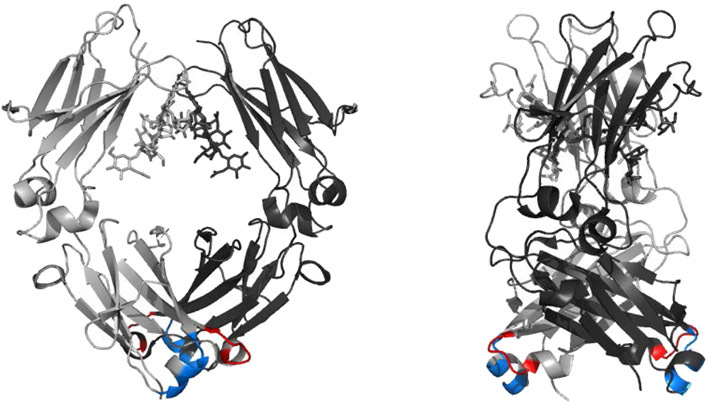
Front and side view of the structure of human IgG1-Fc (PDB-ID 1OQO). The two chains of the homodimeric protein are depicted in light and dark gray, respectively. Each chain comprises a CH2 (top) and a CH3 domain (bottom). The randomized regions in the C-terminal structural loops of the CH3 domains are depicted in red (AB-loop) and blue (EF-loop). For sequence information, see Table 1.

In the present study, we aimed to investigate whether pH-dependent binding sites can also be introduced into antigen binding IgG1-Fc [fragments of immunoglobulin G (Fcab)] molecules. Fcabs contain non-CDR loops, which have been engineered for binding to an antigen (Fig. 1) [8, 9] and thus are promising drugs for therapeutic applications. They combine all antibody properties in a molecule of only approximately one-third of the mass of a full-size antibody: They can (i) trigger immunological clearance mechanisms including antibody-dependent cell-mediated cytotoxicity, (ii) interact with the neonatal Fc receptor (FcRn), and (iii) specifically bind antigens via engineered C-terminal structural loops at the CH3 domains [8–10]. The smaller size of an Fcab might be an advantage in terms of tumor penetration due to increased diffusion. Moreover, its expression in the context of full-size antibodies results in bispecific antibodies. Here, we demonstrate that these artificial binding sites in Fcabs can be engineered for pH-specificity by directed evolution. We have constructed Fcabs with high affinity at pH 7.4 and significantly lower affinity to human epidermal growth factor receptor (Her2-ECD) at pH 6.0. The present study underlines that yeast display is a powerful method for the construction of pH-dependent binding site(s) in different structural contexts.

## 2 Materials and methods

### 2.1 Cloning and library construction

The gene for human IgG1-Fc (including the lower hinge-region, CH2 and CH3 domains) was codon optimized for expression in *Saccharomyces cerevisiae* and cloned into the yeast surface display vector pYD1 (Invitrogen, Carlsbad, CA) by using the *Bam*HI and *Not*I restriction sites, resulting in surface expression of the following fusion construct: Aga2p-GlySerLinker-Xpress-Hinge-CH2-CH3 as described previously [10]. Expression of any C-terminal tags that are present on the original vector was avoided by introducing a stop codon directly after the CH3 domain.

In the parental Fcab clone of this study (H10-03-6), the C-terminal structural loops of the CH3 domains are mutated (Table 1), resulting in binding to Her2 [8]. The following regions are mutated in this clone: Residues 358-362 (Eu numbering system) in the AB-loop and residues 413–415 (including five insertions) and 418–419 in the EF-loop [8]. In the present study, the same positions were randomized by parsimonious mutagenesis [11] using oligonucleotides, which contained 79% of the nucleotide that is present in H10-03-6 and 7% of the respective three other ones. After a second PCR for elongation of the fragment, *S. cerevisiae* EBY100 (Invitrogen) was transformed with the resulting PCR fragment (containing the mutagenized AB- and EF-loop sequences) and the linearized pYD1-Fc vector (missing part of the CH3 domain: AB- and EF-loops and the fragment in between) by using the lithium acetate method [12]. Overlapping regions in the PCR insert and in the linearized vector facilitated homologous recombination in yeast, yielding a library size of 8 × 10^6^ clones. Exact cloning and construction of the library was described previously [10].

**Table 1:** Sequences of selected Fcab clones (P1, P2, and P3), their frequencies in obtained library pools (lib6 and lib6_stringent) pH-dependent interaction of solubly expressed proteins with Her2-positive cells.

Fcab variant	*AB loop*a)	*EF loop*a)	Frequency in respective library (%)		*K*_D_ values (nM)b)		*K*_D_ ratio
			lib6	lib6_stringent	pH 7.4	pH 6	(pH 6/pH 7.4)
Fc-wt	*LTKNQ*	*DKS*xxxxx*RWQQ*	–	–	–	–	–
H10-03-6	YLYGD	PR***H***SARMWRWA***H***	0	0	62 ± 17	105 ± 29	1.7
P1	Y**RH**G**G**	PR***H***SARMWRW**S*H***	0	31	152 ± 17	1201 ± 250	7.9
P2	Y**RN**GD	**A**R***H*T**A**T**MWRW**S*H***	0	15	202 ± 45	c)	c)
P3	YL**S**GD	PR***H***SARMWRW**V*H***	6	6	330 ± 112	c)	c)

a)For structural information about AB- (red) and EF-loop (blue) in the CH3 domains of wild-type Fc (Fc-wt) see Fig. 1. Insertions in the EF-loops of Fcabs are indicated by “xxxxx” in the wild-type sequence. Histidines in the parental clone H10-03-6 and P1, P2, and P3 are depicted in bold green. Mutations with respect to the parental clone are shown in bold violet.

b)Dissociation constants (*K*_D_ values) were obtained by fitting the data points shown in Fig. 4 to a hyperbolic curve. Mean values ± standard deviations for *K*_D_ values from three independent experiments are shown.

c)As the binding curves of P2 and P3 at pH 6.0 did not reach saturation at Fcab concentrations up to 4 μM, reliable *K*_D_ values could not be determined.

### 2.2 Induction of yeast surface expression and selection of Fcab mutants with pH-dependent binding properties

*S. cerevisiae* cultures were grown in SD-CAA medium [20 g/L glucose, 0.1 M KH_2_PO_4_/K_2_HPO_4_, pH 6, 10 g/L (NH_4_)_2_SO_4_, 0.1 g/L l-leucine (all from Sigma, St. Louis, MO), 3.4 g/L yeast nitrogen base, 10 g/L bacto casamino acids (both from Difco, BD, Franklin Lakes, NJ)] at 28 °C over night, followed by sub-cultivation to an OD_600_ of 1 in SD-CAA and cultivation at 28 °C. After 4 h, the yeast suspension was centrifuged and set to an OD_600_ of 1 in SGR-CAA (identical to SD-CAA, but 20 g/L galactose and 10 g/L raffinose instead of glucose, both from Sigma) for induction of surface expression. After 18–20 h of shaking at 20 °C, the cells were harvested by centrifugation. From this step until the flow cytometric sorting, the entire procedure, including all staining and washing steps, was performed in phosphate-buffered saline (PBS)/BSA at either pH 7.4 or 6.0 [2.7 mM KCl, 137 mM NaCl, 10 mM sodium phosphate plus 20 g/L bovine serum albumin (Sigma); either set to pH 7.4 (selection rounds 1, 2, and 5) or to pH 6.0 (selection rounds 3, 4, and 6)]. After two washing steps, the cells were resuspended in 3 nM biotiny-lated Her2-ECD (extracellular domain of Her2, expressed in HEK293 cells and purified by size exclusion chromatography (SEC); biotinylation was done using the EZ-Link Sulfo-NHS-LC-LC-Biotin kit; Thermo Fisher Scientific, Waltham, MA) and incubated at 22 °C for 1 h while shaking. After centrifugation and a washing step, the cells were incubated in PBS/BSA containing 5 μg/mL anti-Xpress-APC [anti-Xpress antibody (Invitrogen) conjugated to allophycocyanin (APC) using the LYNX Rapid APC Antibody Conjugation Kit (AbD Serotec, Kidlington, UK)], 2 μg/mL fluorescein isothiocyanate (FITC) isomer 1-labeled anti-human IgG CH2 domain antibody (anti-CH2-FITC, clone MK 1 A6; AbD Serotec) and streptavidin-R-phycoerythrin (SA-PE, 1:200, Invitrogen). After a final washing step, the cells were sorted by using a fluorescence activated cell sorting (FACS) Aria cell sorter or analyzed on a FACS Canto II (both machines from BD, Franklin Lakes, NJ).

### 2.3 Screening of selected clones and soluble expression in *Pichia pastoris*

After 6 selection rounds 48 clones were sequenced from each of the two resulting libraries. The 10 most enriched Fcab mutants were displayed on yeast again and individually analyzed for binding to Her2-ECD (3 nM) at pH 6.0 and 7.4.

The three best performing Fcab mutants showing the strongest pH-dependence in Her2-binding were sub-cloned into pPICZα for soluble expression in *P. pastoris.* pYD1 vectors comprising the Fcab mutants served as templates for PCRs with primers flanking the Fc gene, followed by *Eco*RI/*Not*I digestion of the resulting PCR products and ligation into pPICZα that had been digested with the same restriction enzymes. Transformation of *P. pastoris* X33 cells with the *Sac*I-linearized vector and induction of soluble expression by addition of methanol was done as described previously [9].

After 5 days of methanol-induced expression, Fcab proteins were purified form the culture supernatants by Protein A affinity chromatography as reported previously [13]. In short, the supernatants were centrifuged and loaded onto a 5 mL HiTrap Protein A HP column (GE Healthcare, Waukesha, WI), followed by washing and elution with 100 mM glycine, pH 3.5. Subsequently, the purified Fcab solutions were dialyzed in PBS (0.2 g/L KCl, 0.2 g/L KH_2_PO_4_, 8 g/L NaCl, and 1.15 g/L anhydrous Na_2_HPO_4_) using a SnakeSkin Dialysis Tubing (10000 MWCO; Thermo Fisher Scientific). For differential scanning calorimetry (DSC) measurements and for analyzing the pH-dependence of binding to Her2, aliquots of the protein samples were also dialyzed in PBS, pH 6.0.

### 2.4 Biophysical characterization by size exclusion chromatography and differential scanning calorimetry

For quality control SEC was performed routinely. Ten micrograms of Fcab protein (either dialyzed in PBS, pH 7.4 or 6.0) were loaded onto a Superdex 200 column (10 mm × 300 mm, GE Healthcare) at 0.75 mL/min in PBS containing 200 mM NaCl as running buffer. Elution was monitored by measuring the absorption at 280 nm [HPLC (Shimadzu LC20) with RI und PDA detectors, software: Lab Solution (Shimadzu)].

For DSC analysis, proteins were diluted to a concentration of 5 μM in PBS, pH 6.0 or 7.4, followed by degassing and analysis by using a VP-DSC Capillary Cell MicroCalorimeter (MicroCal, Northampton, MA). Temperature range and heating rate were 20–110 °C and 1 °C/min, respectively. After subtraction of the buffer baseline, the data were normalized for protein concentration.

### 2.5 Binding to Her2-positive SKBR-3 cells at pH 7.4 and 6.0

SKBR-3 cells were obtained from ATCC and cultivated in McCoy's 5A medium [plus 10% fetal bovine serum (FBS) and 1.5 mM l-glutamine (all from PAA Laboratories, Pasching, Austria)]. Cells were detached by incubation with trypsin/EDTA (PAA Laboratories) and washed twice with PBS/BSA, set to pH 7.4 or 6.0. From these washing steps until the flow cytometric analysis, the entire procedure was done in PBS/BSA at either pH 7.4 or 6.0. The cells were transferred into a 96-well plate (10^5^ cells per well), followed by a 1 h incubation step at 22 °C with Fcab dilutions ranging from 0.24 to 1000 nM. After three washing steps surface bound Fcab was stained with polyclonal R-phycoerythrin-labeled F(ab')_2_ fragment directed against human IgG (anti-hIgG-PE, Sigma) at 4 °C for 30 min, washed again and analyzed on a FACS Canto II. For calculation of the equilibrium dissociation constants (*K*_D_), the measured mean fluorescence intensity (MFImeas) and Fcab-concentrations [Fcab] were fitted to a hyperbolic curve according to the following equation [14].


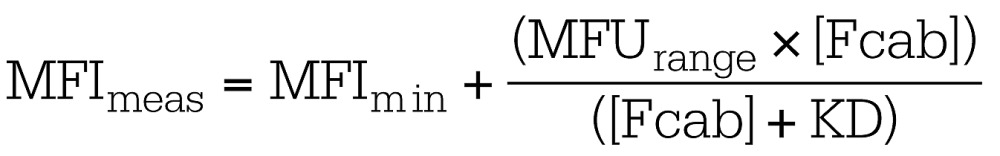


### 2.6 Biolayer interferometry and surface plasmon resonance

Biolayer interferometry was performed by using an Octet QK instrument (ForteBio, Menlo Park, CA). Biotinylated Her2-ECD (4 μg/mL in PBS, pH 7.4) was loaded onto streptavidin-coated Biosensor tips (ForteBio) for 10 min. Subsequently, unspecific binding was blocked by incubating the tips in PBS/BSA, pH 7.4, for 10 min. After measuring the baseline in PBS, pH 7.4 or 6.0, for 10 min, association with Fcab protein at indicated concentrations in PBS, pH 7.4 or 6.0, was monitored, followed by measuring the dissociation, again in PBS at pH 7.4 or 6.0.

Alternatively, Protein A-coated Biosensor tips (ForteBio) were loaded with 200 nM H10-03-6 (in PBS), followed by blocking with PBS/BSA and baseline-measurement (PBS). Finally, association was measured by incubation with indicated concentrations of soluble Her2-ECD in PBS, followed by detection of dissociation in PBS only. For this assay, all incubations were done at pH 7.4.

Interaction between Fc mutants and the neonatal Fc receptor (FcRn) was analyzed by surface plasmon resonance (SPR) by using a BIAcore 3000 instrument (GE Healthcare) as described previously [9]. Briefly, FcRn, which had been expressed in High Five cells and coated onto a CM5 chip, was incubated with 10 μg/mL Fc mutants in PBS, pH 6.0. The pH-dependent dissociation of Fc protein from FcRn was monitored by incubating the chip in PBS, pH 6.0, followed by PBS, pH 7.4.

### 2.7 Molecular dynamics simulation

Based on the X-ray crystal structure of human IgG1-Fc, solved in complex with the ZZ domain of Protein A (Protein Data Bank entry 1OQO), computational design of CH3 domains of wild-type IgG1-Fc was performed. Upon using the FoldX option RepairPDB amino acids exhibiting unfavorable torsion angles, Van der Waals clashes or high energy in the crystal structure were replaced by lower energy rotamers. For computational design of the variant P2 (Table 1), the software LoopX [15] was used for loop reconstruction and side chain modeling in isolated CH3 domains as was described recently by Hasenhindl et al. [16]. For molecular dynamics (MD) simulation the GROMOS11 package was applied [17]. Two systems were created for each the parental clone H10-03-6 and P2 representing protonation of the histidines (Table 1) at pH 6.0 and neutral imidazole side chains at pH 7.0. The four systems were thermalized and simulated for 20 ns. Analysis of the resulting coordinate trajectories was performed as described before [16].

## 3 Results

### 3.1 Library construction

Previously, the Her2-ECD binding Fcab clone H10-03-6 was generated by randomly mutating the structural loops (AB- and EF-loops) at the C-terminal tip of IgG1-Fc and subsequent selection for binding to the antigen [8]. These randomized loop regions included positions 358-362 in the AB-loops and 413–415 (including five insertions) and 418–419 in the EF-loops of the CH3 domains (Table 1).

In the present study, the same loop regions (depicted in Fig. 1) were softly re-randomized by parsimonious mutagenesis, resulting in a library of H10-03-6 variants with an average amino acid mutation rate of 39% with respect to the loop sequences of the parental Fcab clone. Transformation of yeast yielded 8 × 10^6^ independent clones and display of the Fcab library on the surface of yeast was achieved by expressing the following fusion protein: Aga2p-GlySerLinker-Xpress-Fcab.

### 3.2 Selection of Fcabs with pH-dependent binding properties

**Figure 2:**
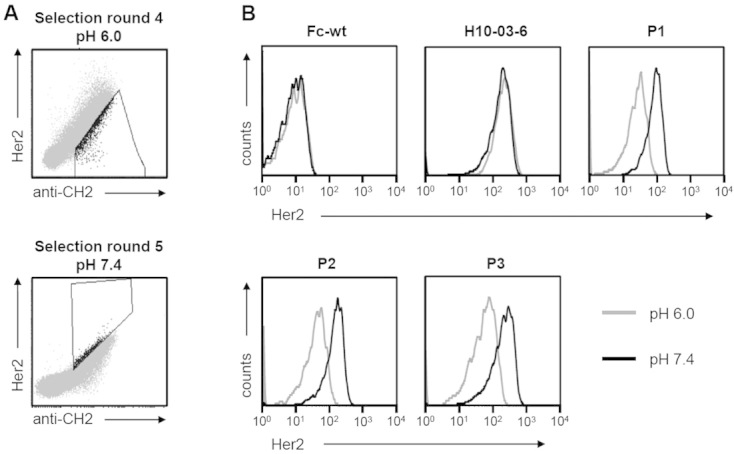
(A) Selection strategy for the generation of pH-dependent binders. A yeast displayed Fcab library was incubated with 3 nM biotinylated Her2-ECD, followed by labeling with SA-PE and anti-CH2-FITC. The incubations were either performed at pH 7.4 (selection rounds 1, 2, and 5) or at pH 6.0 (rounds 3, 4, and 6). If the staining procedure was done at pH 7.4, the cells were subsequently sorted for binding to Her2, whereas incubation at pH 6.0 was followed by selection of non-binders. Exemplarily, the dot plots from selection rounds 4 (top) and 5 (bottom) are shown. The boxes within the plots indicate the gates that were used for sorting. (B) Analysis of the pH-dependence of Her2-binding of selected Fcab mutants. After six rounds of flow cytometric sorting, individual Fcab-clones were analyzed. Fcabs were displayed on yeast and incubated with 3 nM biotinylated Her2, followed by detection of antigen binding with SA-PE and of the surface display level with anti-Xpress-APC (recognizing an N-terminal expression tag located between Aga2p and the Fcab). Incubation steps were either performed at pH 6.0 (gray lines) or 7.4 (black lines). Only Xpress-positive (i.e. displaying) cells were analyzed. In total, 10 Fcabs that have been enriched during selection were tested for pH-dependent Her2 binding. Only the three best performing clones (P1, P2, and P3) are shown.

Recently, this library was used successfully for the generation of stabilized H10-03-6 variants [10]. In the present study, we aimed at selecting variants of Fcab H10-03-6 that bind to the antigen Her2-ECD in a pH-dependent manner, i.e. reduced binding at pH 6.0 compared to pH 7.4. This was done by alternative selection for binding to the antigen at pH 7.4 and non- (or reduced) binding at pH 6.0 (Fig. 2A and Supporting information, Fig. S1, for all selection rounds).

In more detail, in the first two selection rounds incubation with a structurally specific ligand (anti-CH2) and Her2-ECD (3 nM) was done at pH 7.4, followed by selection for simultaneous binding to both, anti-CH2 and to Her2-ECD (Supporting information, Fig. S1). In rounds 3 and 4, the conditions during antigen incubation were changed to pH 6.0 and Fcab clones that showed decreased binding to Her2-ECD were selected. In round 5, the cells were again sorted for interaction with Her2-ECD at pH 7.4, followed by a final sort for non-binding at pH 6.0. In order to investigate whether a more stringent selection strategy is beneficial, two selection campaigns were performed in parallel in rounds 4, 5, and 6: either 1–2% (stringent) or 5–8% (non-stringent) of the population were selected (Supporting information, Fig. S1). In order to avoid selection for Fcab clones that are misfolded at pH 6.0, displayed proteins were selected for binding to a structurally specific antibody (anti-CH2) in all selection rounds. This antibody has been validated for usage in conformationally specific selections in previous studies [13, 17, 18].

After six rounds of selection, the sequences of 48 clones of each library were analyzed. As a first screening, wild-type IgG1-Fc (Fc-wt), the parental clone H10-03-6, as well as the 10 most frequent Fcab clones were displayed on the surface of yeast again and analyzed individually for binding to Her2-ECD (3 nM) at pH 6.0 and 7.4. As expected, Fc-wt does not show any binding at all and interaction of H10-03-6 with Her2 was independent of the pH. However, the three best performing clones (P1, P2, and P3) clearly showed pH-dependent binding to soluble Her2-ECD when being displayed on yeast (Fig. 2B). The loop-sequences of these Fcabs, as well as their frequencies in the library pools after six selection rounds, are shown in Table 1. Importantly, the more stringent selection strategy (with less than 2% of the cells being selected in rounds 3, 4, and 5) turned out to be more efficient for enrichment of pH-dependent binders (Table 1).

### 3.3 pH-dependent interaction of solubly expressed Fcabs with Her2-positive cells

**Figure 3:**
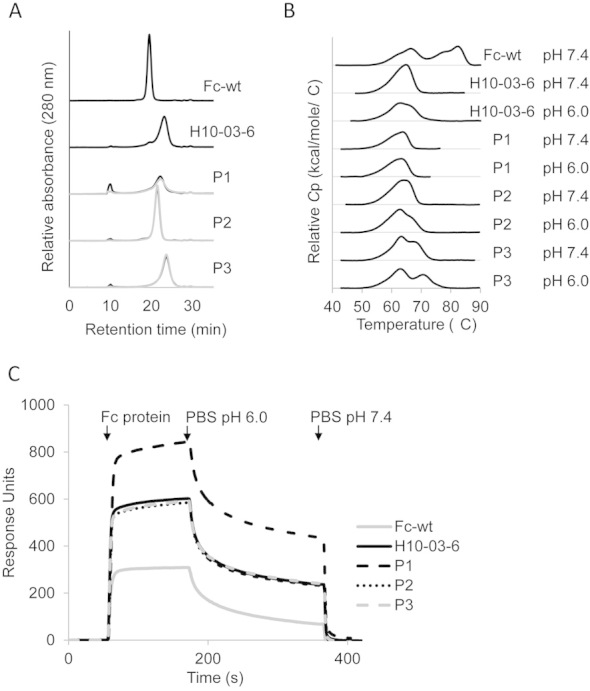
(A) Size exclusion chromatography (SEC) analysis. Ten micrograms of Fc protein [dialyzed in PBS, pH 7.4 (black) or pH 6.0 (gray)] were loaded onto a Superdex 200 column (10 mm × 300 mm, GE Healthcare) at 0.75 mL/min in PBS containing 200 mM NaCl. (B) Differential scanning calorimetry (DSC) analysis. Fcabs were set to a concentration of 5 μM in PBS at pH 6.0 or 7.4. Thermal unfolding was analyzed at a heating rate of 1°C min^–1^. (C) Binding of Fcabs to FcRn. FcRn was immobilized onto an SPR chip. The coated chip was floated with 10 μg/mL Fcab (in PBS pH 6.0). Finally, pH-dependent dissociation was monitored in PBS pH 6.0, followed by PBS pH 7.4.

The three most promising Fcab clones shown in Fig. 2B (P1, P2, and P3) were sub-cloned for soluble expression in the yeast *P. pastoris.* First, purified Fcabs were analyzed by SEC for quality control. As reported previously [10], the lead Fcab of the present study (H10-03-6) shows an altered SEC profile with an elevated retention time and peak broadening compared to Fc-wt (Fig. 3A). Moreover, a shoulder at the main elution peak, as well as a small peak at a retention time of approximately 10 min, indicate some aggregation. SEC analysis of P1 and P3 showed comparable elution characteristics, except for some minor differences in the retention times and increased detection of high molecular weight aggregates in the P1 sample. However, the SEC profile of Fcab P2 was clearly improved compared to the parental clone H10-03-6. The elution peak was narrowed, the retention time was more wild-type like and the significant reduction of the shoulder on the left of the peak indicates less aggregation. Generally, elution profiles of Fcabs dialyzed in PBS, either pH 6.0 or 7.4, were almost identical (Fig. 3A).

**Figure 4:**
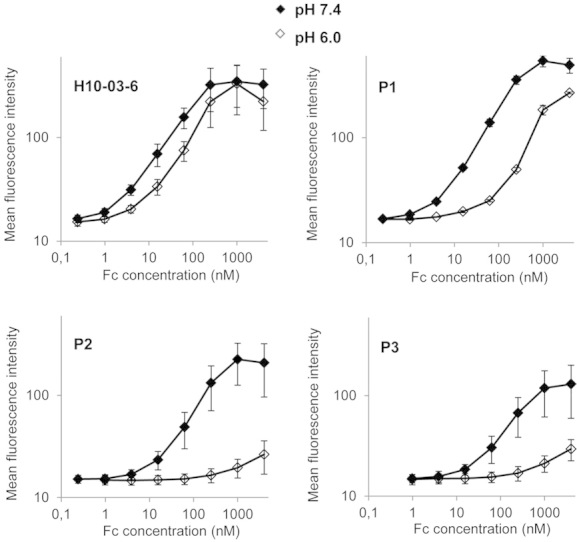
pH-dependent binding of Fcabs to Her2 positive cells. The Her2 positive cell line SKBR-3 was incubated with various Fcab concentrations. Subsequently, surface bound Fcab was detected with polyclonal R-phycoerythrin-labeled F(ab')_2_ fragment directed against human IgG. The incubations were performed at either pH 6.0 (empty) or 7.4 (filled symbols). Mean values ± standard deviations from three independent experiments are shown.

Next, it was investigated whether the pH-dependent interaction between yeast displayed Fcabs and soluble Her2-ECD (described above, Fig. 2B) is also observed between soluble Fcabs and a Her2 positive cell line. The cells were incubated with various Fcab concentrations, followed by labeling of surface bound Fcab with anti-hIgG-PE and flow cytometric detection. The entire staining procedure was either performed at pH 6.0 or 7.4. The parental clone H10-03-6 showed a slight pH-dependence with a *K*_D_ ratio of 1.7 [*K*_D_ at pH 6.0 (105 nM) divided by *K*_D_ at pH 7.4 (62 nM); Fig. 4 and Table 1]. Although binding at pH 7.4 was slightly weakened for all three selected Fcab variants (Table 1), the pH-dependence could be markedly enhanced. For the clone P1 the *K*_D_ ratio was increased to 7.9 (Fig. 4 and Table 1) and in the case of Fcab clones P2 and P3 the affinity reduction at pH 6.0 was even more pronounced, making it impossible to calculate reliable *K*_D_ values (Table 1). Nevertheless, the binding curves of P2 and P3 clearly demonstrate the strong pH-dependence of Her2 binding (Fig. 4).

In order to investigate whether the observed pH-dependence in Her2 binding is caused by unfolding of the Fcab at pH 6.0, the thermal stabilities of the Fcabs were analyzed at both pH 7.4 as well as pH 6.0 by DSC. In recombinant wild-type IgG1-Fc expressed in *P. pastoris*, three endothermic transitions were observed reflecting unfolding of the CH2 (*T*_m_1 at 66 °C) and CH3 domains (*T*_m_2/*T*_m_3 at 78 and 83 °C) [9]. Increasing the temperature to the endothermic transition of the CH3 domains leads to irreversible denaturation of IgG1-Fc and Fcabs.

The DSC profile of H10-03-6 and P1 only showed one broad transition at ∼65 °C, demonstrating that the CH3 domain was strongly destabilized and that its *T*_m_ values shifted toward the one of CH2 (Fig. 3B) [13]. In comparison with the parental clone, the thermal stability of the CH3-domain of P3 was increased. Importantly, the thermal unfolding of P1, P2, and P3 at pH 7.4 and 6.0 revealed no striking differences (Fig. 3B), demonstrating that both the fold and stability of these Fcabs do not change within pH 6.0 and 7.4. This is also reflected by SEC elution profiles at pH 6.0 (not shown), which are almost identical to those obtained at pH 7.0 (Fig. 3A).

Next, we probed the pH-dependent interaction of Fcabs P1, P2, and P3 with the neonatal Fc receptor (FcRn) by SPR. Typically, FcRn binds strongly at pH 6.0 but shows weak binding at pH 7.4 [19, 20]. After incubating the FcRn-coated SPR chip with Fcab solution at pH 6.0, the pH-dependent dissociation was analyzed by floating the chip with PBS, pH 6.0, followed by PBS pH 7.4. Importantly, all analyzed Fcabs showed the typical pH-dependent FcRn-binding behavior with slow dissociation at pH 6.0 but rapid release at pH 7.4 (Fig. 3C). Thus, the interaction of the Fcabs P1, P2, and P3 with FcRn is stronger at pH 6.0, whereas their affinity to Her2-ECD is higher at pH 7.4. These findings together with the DSC data (Fig. 3B) clearly suggest that the reduced interaction of Fcabs P1, P2, and P3 with Her2-ECD at pH 6.0 compared to pH 7.4 is not mediated by partial unfolding and/or reduced stabilities of the evolved Fcab mutants at this pH value.

### 3.4 Interaction of immobilized Her2 with soluble Fcabs

Next, we measured the interaction of immobilized Her2 with soluble Fcab by biolayer interferometry. Biotinylated Her2 was loaded onto streptavidin-coated tips, followed by blocking with PBS/BSA. Subsequently, binding of soluble Fcab was analyzed at pH 7.4 or 6.0. In contrast to the experiments described above, in this experimental setting Fcab P2 did not show a considerably higher pH-dependence in Her2-binding compared to H10-03-6 (not shown). Both Fcabs showed slightly stronger binding at pH 7.4 compared to pH 6.0. At first sight, this observation was surprising but most probably reflects the avidity effect, which is caused by the interaction of homodimeric IgG-Fc with two immobilized Her2 molecules and thus abolishes the pH-dependent interaction. Similar observations have been made by others [7] and are discussed below.

### 3.5 Molecular dynamics simulation of P2 at pH 6.0 and 7.0

It was interesting to see that all three pH-dependent Fcab mutants contained mutations next to the histidines in the EF-loop region of the parental clone. The mutant P1 had one additional His in the AB-loop (Table 1). It is reasonable to assume that the mutations close to the histidines modulate the microenvironment and the p*K*_a_ of the existing histidines. In order to get a better understanding about the role of the histidines in the EF-loop, the structures of the CH3 domain of the parental clone H10-03-6 and the mutant P2 were simulated at pH 6.0 (protonation of His) and pH 7.0 (neutral His side chains). In a previous publication, we could demonstrate that the isolated CH3 domain can be used for MD simulations since the C-terminal loops of one CH3 domain within homodimeric IgG1-Fc do not significantly interact with the loops of the other CH3 domain and the individual contributions to the antigen binding site seem to be independent [21].

The results obtained for the four described systems suggest almost identical structural stability and domain dynamics. Analysis of the atom-positional root-mean-square deviations of all atoms (RMSD) showed no constant increase of the structural deviations from the reference structure in any of the systems. Root-mean-square fluctuations (RMSF) of backbone atoms were determined and revealed distinct fingerprints of framework- and loop regions that did not differ significantly in the analyzed systems (Supporting information, Fig. S2). Determination of the average number of hydrogen bonds formed at every point in time during the simulation did not reveal any significant differences between the protonated and neutral states either. Moreover, the average solvent accessible surface area of the binding loops was similar in all four systems.

## 4 Discussion

This study demonstrates that artificial binding sites of Fcab molecules (i.e. C-terminal structural loops in CH3 domains) can be engineered for pH-dependent antigen binding. We showed that yeast display, which has already been applied successfully for a variety of tasks in protein engineering [22–25] can also be used for directed evolution of pH-dependent binding sites. As a model system, the well described Her2-specific Fcab clone H10-03-6 was chosen as lead protein [8]. We used a library that was constructed by parsimonious mutagenesis of the AB- and EF-loops of the CH3 domains and has already been successfully used for the selection of stabilized variants of H10-03-6 [10]. In this library, 5 positions in the AB-loop, as well as 10 positions in the EF-loop are mutated. Recent studies have demonstrated that mutations at positions R416 and W417 in the EF-loop of the CH3 domain have negative impacts on the fold and stability of the Fc protein [18, 26]. Therefore, these positions were excluded from mutagenesis.

The directed evolution strategy employed in this study was based on alternating selections for binding at pH 7.4 and non-binding at pH 6.0. Indeed, this procedure enabled enrichment of Fcab mutants, which show differential binding at these two pH values. Importantly, we could show that the differential binding was not caused by conformational changes of the Fcab variants at pH 6.0. Both MD simulations (Supporting information, Fig. S2) as well as DSC experiments (Fig. 3B) suggested very similar conformations at both pH values. Moreover, the Fcabs retained their pH-dependence for FcRn-binding, i.e. strong binding at pH 6.0, but very weak binding at pH 7.4. Thus, interaction of these evolved Fcab mutants with FcRn was stronger at pH 6.0, whereas the affinity to Her2 was higher at pH 7.4 (Fig. 3C), clearly demonstrating that the measured pH-dependence of antigen binding cannot be a result of partial unfolding at a certain pH.

Surprisingly, when binding of soluble Fcabs to immobilized Her2 was analyzed by biolayer interferometry, the engineered Fcab P2 did not show markedly increased pH-dependency compared to the parental clone H10-03-6. One possible explanation for this phenomenon is that avidity abrogates the pH-dependency. When Fcabs were displayed on yeast and the interaction with soluble monomeric Her2-ECD was measured, a clear shift in binding intensity could be detected when comparing pH 7.4 with pH 6.0. This pH-dependency was also observed when soluble Fcab was titrated on Her2-positive SKBR-3 cells. However, when the interaction between soluble Fcab and Her2 immobilized onto biolayer interferometry tips was analyzed, hardly any pH-dependence was detected. Principally in both assays (binding to Her2-positive cells or to Her2-coated tips), the homodimeric Fcab molecule could potentially interact with two Her2 molecules. However, the density of Her2 on the surface of SKBR-3 cells is expected to be much lower than on artificially coated biolayer interferometry tips. Thus, the avidity effect will be much more pronounced in the biolayer interferometry assay. In fact, similar results were also reported for the pH-dependent interaction between Fc-binding Sso7d and IgG-Fc [7]. In an ELISA assay, monomeric soluble Sso7d bound to immobilized IgG-Fc in a pH-dependent manner. The pH-dependence was also observed when soluble dimeric Fc was incubated with yeast-displayed Sso7d. However, when Sso7d was immobilized onto ELISA plates and soluble IgG was added, pH-dependence was not observed any more. These results are very similar to our observations. In both studies, pH-dependence is only observed if the monomeric interaction partner is added in soluble form, or if the surface density is so low that the soluble dimeric molecule will mostly just bind to one molecule at a time. Thus, to sum up, avidity effects might abrogate the pH-dependence of monovalent interactions. Importantly, Gera et al. [7] observed this phenomenon for both Sso7d-variants that have been engineered for pH-dependent binding by either directed evolution in a similar way to our study or by histidine-scanning. This shows, that the possible abrogation of pH-dependent binding by avidity effects is not related to the method that has been chosen for constructing the pH-dependent binding site.

In most of the studies that have been conducted so far, pH-dependent binding sites have been engineered by histidine substitutions [3–6]. The rationale behind this approach is the fact that the histidine side chain has a p*K*_a_ of approximately 6. This means that it is protonated by shifting the pH from neutral to mildly acidic conditions. We hypothesized that a library, which has been constructed by parsimonious mutagenesis, should also cover all histidine mutations. In the initial library, the randomized positions contained approximately 79% of the original nucleotide found in the parental lead Fcab, and 7% of the three other ones, respectively. At the last position of the codons, 86% of the original and 14% of one other nucleotide were present (86%G/14%T or 86%T/14%G or 86%C/14%G). Adenine was excluded in order to minimize the frequency of stop codons. Thus, even if all three nucleotides had to be changed in order to reach a His-codon, this codon should have a frequency of 0.07 × 0.07 × 0.14 = 6.8 × 10^–4^ in the library. Given the library size of 8×10^6^ individual clones, about 5000 Fcab clones (8 × 10^6^ × 6.8 × 10^–4^) would be expected to have this particular His-mutation at the respective position. For positions, where only one or two nucleotide changes were necessary in order to reach the His-codon, this number would even be markedly higher.

Surprisingly, the selected Fcab mutants P2 and P3 did not contain a single histidine-mutation (Table 1) and in the mutant P1 there was only one His-substitution. However, it should be noted that although the parental clone H10-03-6 did not bind to Her2 in a pH-dependent manner, it contained two histidine residues in the mutated part of the EF-loop (Table 1). Strikingly, all three pH-dependent Fcab mutants contained a mutation next to the histidine located at the last position of the randomized EF-loop region (Table 1). Moreover, in P2 there was another mutation next to the second histidine. The accumulation of mutations in the close proximity of existing histidines suggests that the formation of the pH-dependent switch might be facilitated by a change in the microenvironment of these histidines.

This finding is consistent with the recent study by Gera and colleagues, where yeast display was used to engineer pH-dependent Fc-binders based on the hyperthermophilic Sso7d scaffold [7]. Although not specifically discussed by these authors, histidines were hardly detected in the selected sequences. Remarkably, the mutant Sso7d-ev-hFc, which was characterized in more detail and which clearly showed stronger binding at pH 7.4 compared to 4.5, did not contain a single His-residue in the engineered binding site. This could be explained by the presence of histidine(s) in the epitope or by the presence of electrostatic interactions involving other amino acids than histidines.

This study demonstrates that yeast display is a powerful method for engineering pH-dependent binding sites. This was also demonstrated in a recent study, where Sso7d-based binders were engineered for pH-dependent interaction with human IgG-Fc [7]. The fact that yeast display based selections enabled the construction of pH-dependent binding sites in two different scaffolds highlights the potency of this technology for this task in protein engineering. In contrast to our expectations, in both studies histidines were hardly selected in the binding sites. However, we saw an enrichment of mutations in the close proximity of existing histidines. This suggests that in certain cases, for example, if there are already histidines in the interaction interface (on either interaction partner), mutations, which change the microenvironment of existing histidines might be more efficient for engineering of pH-dependent binding sites than histidine-mutations themselves.

## Abbreviations

APC: allophycocyanin
DSC: differential scanning calorimetry
FACS: fluorescence activated cell sorting
Fc: crystallizable fragment
Fc-wt: recombinant wild-type Fc
FITC: fluorescein isothiocyanate
H10-03-6: Her2 binding lead Fcab
Her2-ECD: extracellular domain of human epidermal growth factor receptor 2
hIgG: human immunoglobulin G
IgG1y: immunoglobulin G class 1
>PBS: phosphate-buffered saline
PE: phycoerythrin
SA: streptavidin
SEC: size exclusion chromatography
SPR: surface plasmon resonance

## Notes

Supporting information available online
